# Inhibition of telomerase activity by HDV ribozyme in cancers

**DOI:** 10.1186/1756-9966-30-1

**Published:** 2011-01-06

**Authors:** Yingying Lu, Junchao Gu, Dachuan Jin, Yanjing Gao, Mengbiao Yuan

**Affiliations:** 1 Department of Medicine, Beijing Friendship Hospital affiliated to Capital Medical University, Beijing, 100050, PR China; 2 Department of Digestive disease, Qilu Hospital affiliated to Shandong University, Jinan, Shandong Province, 370045, PR China

## Abstract

**Background:**

Telomerase plays an important role in cell proliferation and carcinogenesis and is believed to be a good target for anti-cancer drugs. Elimination of template function of telomerase RNA may repress the telomerase activity.

**Methods:**

A pseudo-knotted HDV ribozyme (g.RZ57) directed against the RNA component of human telomerase (hTR) was designed and synthesized. An in vitro transcription plasmid and a eukaryotic expression plasmid of ribozyme were constructed. The eukaryotic expression plasmid was induced into heptocellular carcinoma 7402 cells, colon cancer HCT116 cells and L02 hepatocytes respectively. Then we determine the cleavage activity of ribozyme against human telomerase RNA component (hTR) both in vitro and in vivo, and detect telomerase activity continuously.

**Results:**

HDV ribozyme showed a specific cleavage activity against the telomerase RNA in vitro. The maximum cleavage ratio reached about 70.4%. Transfection of HDV ribozyme into 7402 cells and colon cancer cells HCT116 led to growth arrest and the spontaneous apoptosis of cells, and the telomerase activity dropped to 10% of that before.

**Conclussion:**

HDV ribozyme (g.RZ57) is an effective strategy for gene therapy.

## Background

Immortalized and malignant tumor cells are characterized by unlimited cell proliferation and programmed cell death (apoptosis). It has been demonstrated that malignant transformation occurs when the telomerase in normal cell is activated [1,2].

Telomerase activity is found in almost all malignant tumors [3]. Human telomerase RNA (hTR) is associated with the activity of telomerase, immortalized cancer cells retain the highest level of hTR [4,5]. In recent years, hammerhead ribozymes were used to inhibit the telomerase activity by targeting the template region of telomerase RNA in malignant tumors [6,7]. Yet, there is no report about HDV ribozyme for inhibition of telomerase activity.

Ribozymes are catalytic RNA molecules which can be designed to specially cleave a target RNA sequence by incorporating the flanking sequence complementary to the target[8]. Like other ribozymes, HDV ribozyme has this property. So it may have a potential application in gene therapy in which an engineered ribozyme is directed to inhibit gene expression by targeting a specific mRNA molecule.

As hepatocellular carcinoma is often associated with the infection of HBV and HDV, The facts that HDV ribozyme derived from HDV and that pathogen naturally infects and replicates in hepatocytes suggest that it can be used to control gene expression in human cells. The HDV ribozyme is active *in vitro *in the absence of any proteins, it is the only known example of a catalytic RNA associated with an animal virus. there are no known homologues of HDV ribozymes, and sequence variation of the HDV ribozymes in clinical isolates is minimal.

Then we imagine whether HDV ribozyme can be used to inhibit hepatocellular carcinoma. In the present study we designed a HDV ribozyme against RNA component of human telomerase in hepatocellular carcinoma cell lines, as well as in normal hepatocytes and other cancers, then examined the function of the HDV ribozyme and the effects of developing the HDV ribozyme as a tool of cancer gene therapy

## Methods

The bel7402, HCT116 cells were given by Department of molecular Biology, Shandong University, DNA of HDV ribozyme was synthesized by Shanghai Biosun Sci&Tech. Co. LTD. Recombinant plasmid pBBS212 containing hTR gene was provided by Geron Company.

### Design and synthesis of HDV ribozyme

**Figure 1:**
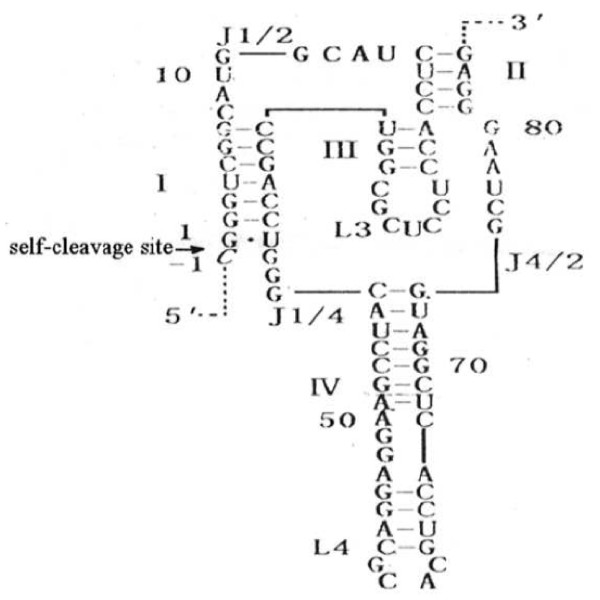
**Structure of antigenomic ribozyme of HDV (g.RZ 1/84)**.

It was demonstrated that antigenomic ribozyme of HDV (g.RZ 1/84) is composed of 84 nucleotides[9]. It composed four stems (P1-P4), two loops and three junctions. As seen in Figure 1.

gRZ.1/84 can cleave 8-13 nt substrate by inter-molecular cleavage [10], the substrate must integrate with P1 stem of HDV ribozyme through base-pairing before cleavage, only 7 nt base pairing are needed, then the cleavage can occur. In P1 stem G.U wobbling pair is essential for the activity of gRZ.1/84 and cannot be changed. The other 6 nucleotides can be changed, but the change must keep Waston-Crick pairing to substrate [11-13]. P4 stem isnot essential and can be deleted for easier access of ribozyme to substrate [14]. The activities of modified ribozyme do not decrease, but sometimes increase [15,16].

We chose 12-84 nt of g.RZ 1/84, deleted 16 nt from P4 stem, and changed 6 nt of P1 stem from CCGACC to GGUUGA, only keeping G.U wobbling pair, to meet the need of cleavage of telomerase. We called the new ribozyme g. RZ57. The double-sranded DNA of g. RZ57 was synthesized with ApaΙ and HindIII protruding ends. Their sequences are as follows: 5' AGCTT GGGAC CACCA CCACG CGGAC GCAAG AAGGG CAAGC GGCAA CGCAA GGCAA AGGGACCC CCC 3' and 5' A CCCTG GTGGT GGTGC GCCTG GCTGG TCCCG TTCGC CGTTG CGTTC CGTTT CCCTG GG GGG 3'.

**Figure 2:**
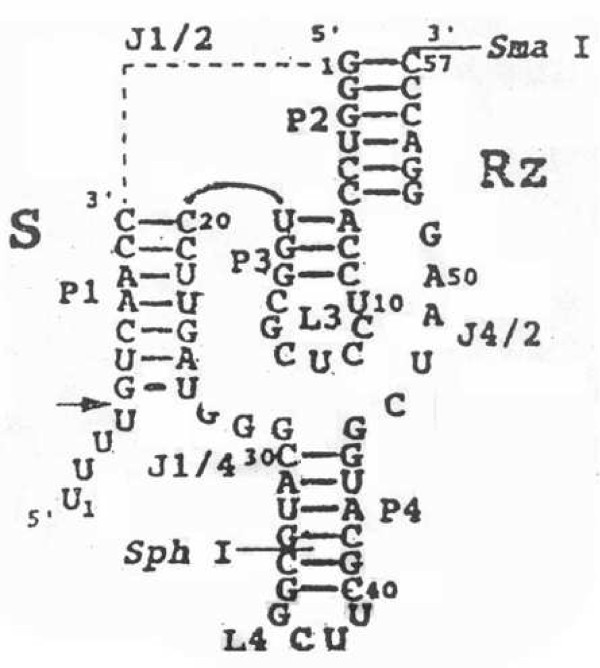
**The secondary structure of HDV ribozyme annealed to the hTR, the target site GUC is just above the arrow, the arrow indicates the site of cleavage**.

The predicted secondary structure of g. RZ57 are seen in Figure 2.

After annealing, the fragments were ligated to ApaΙ and HindIII co-digested PGEM- 7Zf (+). This plasmid was denoted as PGEM.RZ. It is the in vitro plasmid of HDV ribozyme. We also ligated the fragments to ApaΙ and HindIII co-digested pcDNA3.1 (+). This plasmid was denoted as pcDNA.RZ. It is the eukaryotic expression plasmid of HDV ribozyme.

### Telomerase RNA plasmid construction

We cloned a portion of hTR component containing a telomeric template element using

RT-PCR. In normal conditions, only inhibition of the template region can lead to the inhibition of telomerase activity. we clone a portion ranging from 19 nt to 88 nt of hTR. There are 14 template regions (GUC sequence) in this portion. We chose one site (47-50 nt) as cleavage site. Primers for RT-PCR were as follows: 5'CTGGG AGGGG TGGTG GCCAT 3'(upstream) and 5'GGAGC AAAAG CACGG CGCCT 3' (downstream). 70 nt product is amplified by 25-30 cycles of PCR(50°C 30 min; 94°C 2 min; 94°C 30 s, 55°C 30 s, 72°C 1 min). The purified products were cloned into PGEM-T plasmid. The resulting plasmid is denoted as PGEM.hTR. The obtained human telomerase component was verified by DNA sequencing.

### In vitro cleavage reaction by ribozymes

Plasmid PGEM.RZ was linerized by SmaI, and PGEM.hTR by EcoRV respectively. Then in vitro transcription kit Riboprobe^® ^system- Sp6/T7 P1460 was used to transcript plasmids. We got a 80 nt RNA fragment of HDV RZ(part is carrier fragment), and a 90 nt RNA fragment of hTR (part is carrier fragment).

After hTR was radioactively labeled, we mixed the ribozyme and substrate RNA(molar ratio 2.5:1, 5:1, 10:1, 20:1 respectively) at different temperature in a 20 μl reaction volume containing 50 mM Tris-HCl(PH 7.5) and 1 mM EDTA.

At different time 5 μl mixture was taken to electrophorese on 5% agorose gel, and the results were quantitatively analyzed by autoradiography to calculate the cleavage rates.

### Transfection of bel-7402 and HCT116 cells

The bel7402, HCT116 cells (5 × 10^4^) were seeded in 6-well plates, a day before transfection. Lipofections of heptocellular carcinoma 7402 cells, colon cancer cells HCT116 and normal human heptaocyte L02 with both the 10 μg pcDNA.RZ vector and PGEM-7Zf (+) were performed according to the protocol recommended by the manufacturer (Life Technologies, Inc). After 24 h, 48 h, 72 h, all cells were scored for apoptosis, telomerase activity assay and respectively.

### Telomerase activity assay

Cellular telomerase activity was measured with TRAP-ELISA kit (Roche Diagnostics GmbH). The cells (about 10^5^-10^6^) were collected and washed twice by PBS, lyzed in 200 μl of cell lysis buffer, incubated at 4°C for 30 min, then centrifuged at 16,000 rpm for 10 min.

Telomerase activity was determined before and after the induction of ribozyme plasmid. The telomerase activity A was semiquantified photometrically at 450 nm and 690 nm. A = A_450_-A_690_. The results were tested by t test.

### Northern blot analysis

Twenty micrograms of total RNA was loaded on 1% agarose/formaldehyde gel, electrophoresed, and then mounted on a nylon membrane by capillary transferA single - strand probe was generated by RT-PCR of a 184 bp fragment by of hTR cDNA by digestion of Recombinant plasmid pBBS212 containing hTR gene (provided by Geron Company) with EcoRI. The purified fragment was mixed with 15 pmol of dNTP and 25 Ci of [a- 32P] dCTP (NEN Life Sciences) in 20 mM Tris-HCl, 50 mM KCl, pH 8.4, 1.5 M MgCl2, containing 0.2 g/L hTR forward primer 5'-CTGGG AGGGG TGGTG GCCAT-3') and 2.5 U of Ex Taq DNA polymerase (TaKaRa Biotech, Shiga, Japan).

Amplification was carried out with 34 cycles of denaturation at 94°C for 30 seconds,

annealing at 60°C for 30 seconds, and extension at 72°C for 1 minute. After purification, the hTR probes were heated at 100°C for 5 minutes and immediately added to hybridization reaction.

### Cell cycle and apoptotic rate analysis

Growing cells (about 2 × 10^6^) were collected and fixed with 70% cold ethanol for at least 12 h, then were stained by propidium iodide. Cells were analyzed for the cell distribution and apoptotic rate by DNA analysis using FCM.

### Statistical Analysis

The student's test and X^2 ^test were used to evaluate the statistical significance of the results. All analyses were performed with SPSS statistical software.

## Results

### In vitro cleavage reaction

**Figure 3:**
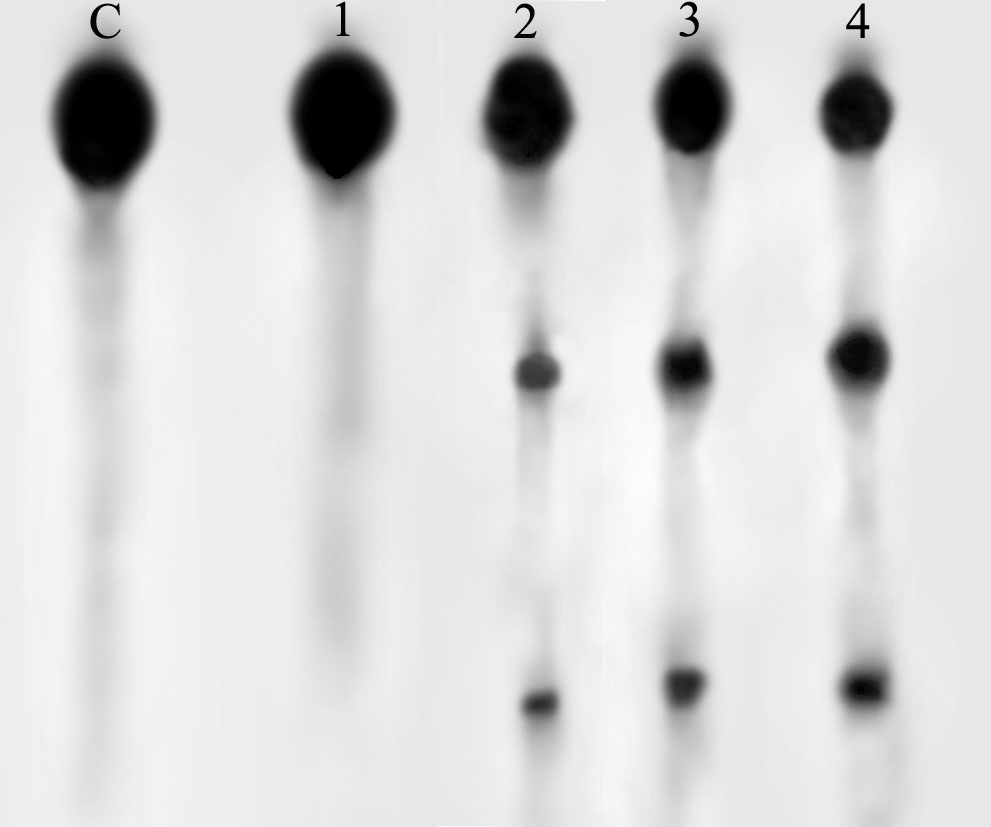
***In vitro *cleavage in a mixture of the RNA substrate and RZ at molar ratio 5:1 and at 45°C, after 0,1, 2, 3 hours of incubation respectively**. (lanes 1-4, lane C is the control lane; 1. hTR+ RZ (0 h); 2. hTR+ RZ(1 h); 3. hTR+ RZ (2 h). 4. hTR+ RZ (3 h))

According to this research, the most suitable temperature for HDV RZ cleavage is 45°C, a little lower than hammerhead RZ (55°C). RNA will degrade higher than 45°C. The most suitable molar ratio is 5:1 and the most suitable cleavage time is two hours. The maximum cleavage ration is 70.4%. Lengthening the reaction time or increasing the RZ/hTR ratio cannot increase the cleavage ration. In the case of control RZ, no obvious catalytic activity was detected. One cleavage process was shown at molar ratio 5:1 and at the temperature 45°C in Figure 3.

### The telomerase activity

Cellular telomerase activity of eukaryotic bel7402-RZ, HCT116-RZ and L02-RZ are shown in table 1. The telomerase activity of bel7402-RZ cells dropped continuously. It dropped to 10% of that before after 72 hours. While the L02-RZ cells almost have no change, as seen in table 1.

**Table 1:** The telomerase activity of ribozyme tranfected cells

	0 hr	24 hr	48 hr	72 hr	96 hr
bel7402-RZ	0.87 ± 0.09	0.59 ± 0.05	0.28 ± 0.06*	0.08 ± 0.01*	0.08 ± 0.01*
HCT116-RZ	0.84 ± 0.10	0.65 ± 0.07	0.32 ± 0.08*	0.13 ± 0.05*	0.10 ± 0.03*
L02-PGEM	0.85 ± 0.09	0.84 ± 0.10	0.81 ± 0.06	0.80 ± 0.05	0.78 ± 0.04
L02-RZ	0.87 ± 0.09	0.80 ± 0.12	0.78 ± 0.09	0.75 ± 0.11	0.72 ± 0.07
bel 7402- PGEM	0.87 ± 0.09	0.81 ± 0.07	0.82 ± 0.03	0.83 ± 0.04	0.82 ± 0.04
HCT-PGEM	0.89 ± 0.11	0.85 ± 0.14	0.80 ± 0.08	0.77 ± 0.06	0.71 ± 0.10

*P < 0.01

### Northern blot analysis

**Figure 4:**
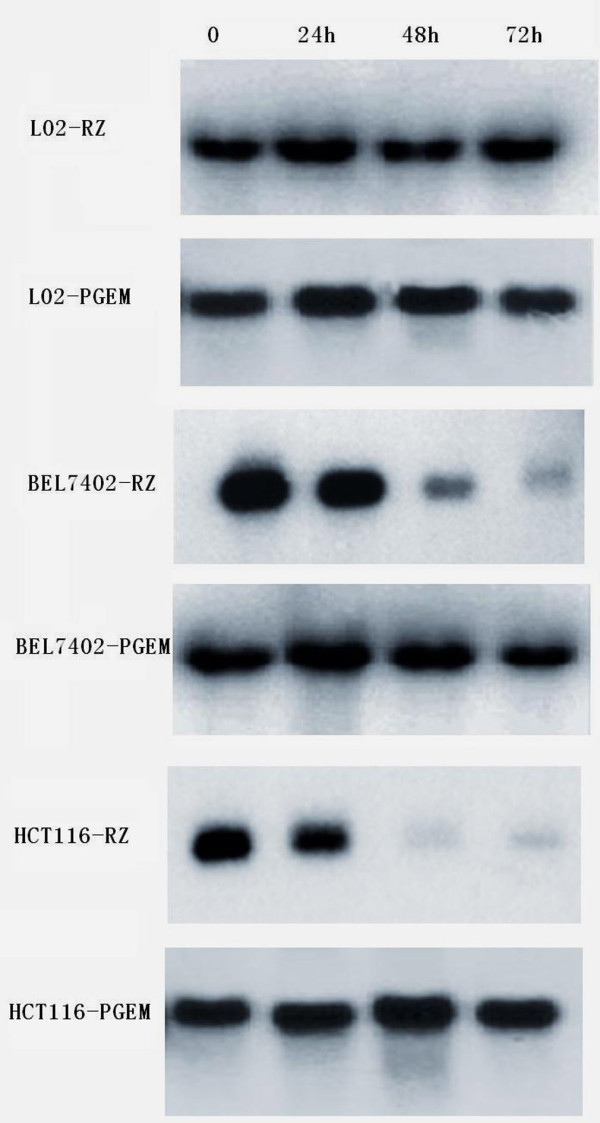
**Time course of Northern blot analysis of hTR RNA in different cell lines after transfection 0, 24, 36, 72 hours respectively**.

Ribozyme transfected bel7402 cells and HCT116 cells showed decrease of hTR RNA. In ribozyme transfected bel7402 cells, the uncut hTR decreased to 1/25 of the original, in HCT116 cells, the uncut hTR decreased to 1/20 of the original; while the others did not obviously decrease (seen in Figure 4).

### Cell cycle distribution and apoptotic rate of 7402 cells

**Figure 5:**
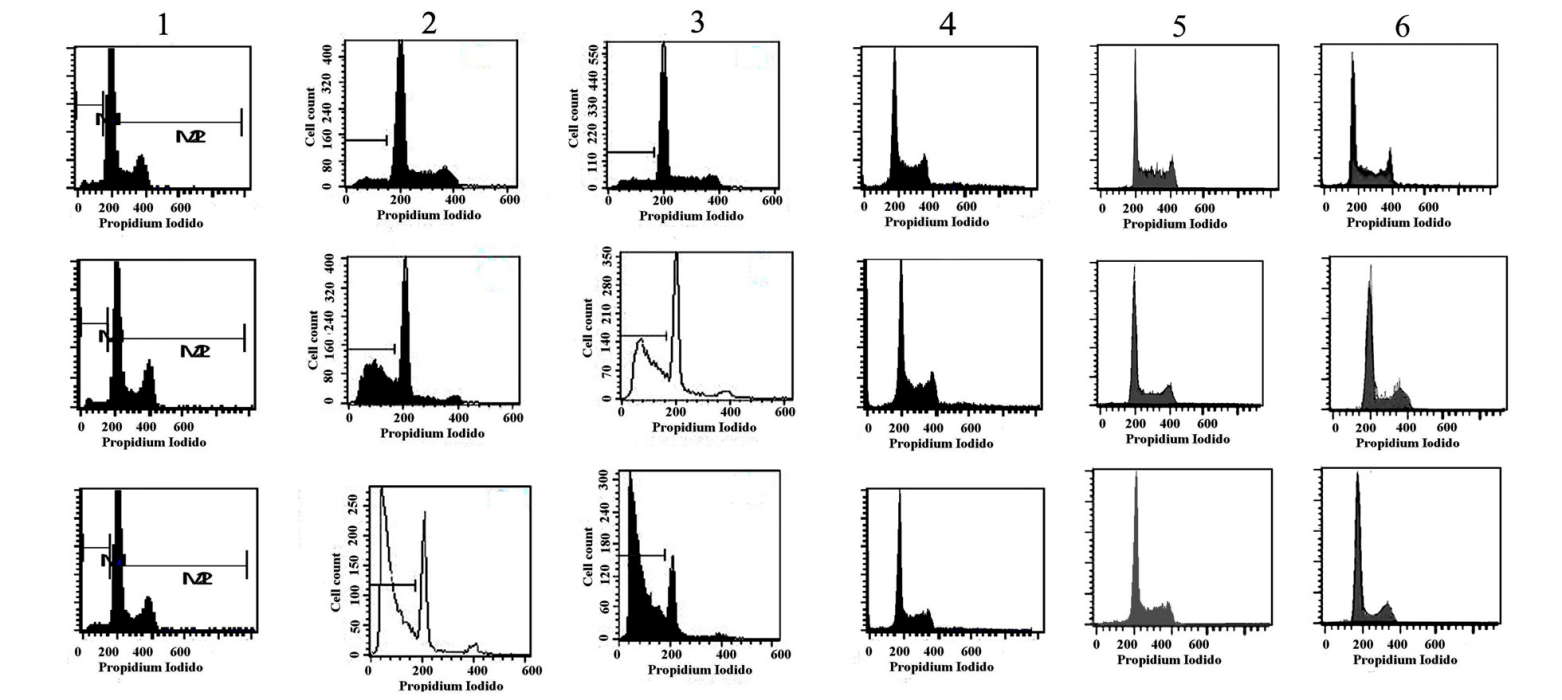
**Apoptotic rate of ribozyme-transfected and PGEM vector transfected cells (1-6)**. 1 bel 7402 +PGEM-7Zf (+); 2. bel 7402 +RZ; 3. HCT116+RZ; 4. HCT116+ PGEM-7Zf (+); 5. L02+RZ; 6. L02+ PGEM-7Zf (+)

Ribozyme transfected 7402 cells and HCT116 cells displayed an increased percentage of cells in the G0/G1 phase and apoptotic rate, as compared with other cell lines, The results are shown in table 2 and Figure 5.

**Table 2:** Cell cycle distribution and apoptotic rate in ribozyme-transfected and control cells

Cell line	Cell cycle distribution (%)			Apoptotic rate (%)		
	
	G0/G1	S	G2/M	24 hr	48 hr	72 hr
L02-RZ	50.8 ± 4.9	28.1 ± 5.9	21.1 ± 3. 7	1.7 ± 0.1	2.0 ± 0.2	2.3 ± 0.4
bel 7402-RZ	71.7 ± 6.1	12.1 ± 2.0	17.0 ± 2.9	14.3 ± 2.3	35.2* ± 4.9	75.5* ± 6.5
HCT116-RZ	56.2 ± 5.5	17.5 ± 2.5	26.3 ± 3.7	9.6 ± 1.9	20.4* ± 3.4	59.7* ± 5.7
bel 7402-PGEM	58.0 ± 5.0	19.2 ± 2.7	22.6 ± 3.0	0.8 ± 0.05	2.6 ± 0.7	4.3 ± 1.1
L02-PGEM	55.0 ± 6.9	27.8 ± 4.8	7.2 ± 2.3	2.3 ± 0.9	5.8 ± 1.0	8.6 ± 0.7
HCT116- PGEM	60.1 ± 10.2	18.3 ± 7.4	22.6 ± 3.7	2.5 ± 0.3	3.4 ± 0.7	5.2 ± 0.6

## Discussion

Telomerase activity increases in most malignant tumors. To inhibit the telomerase activity is a new method for tumor therapy [17]. Human telomerase RNA is closely associated with telomerase activity. The template region is crucial for enzyme activity, and this site is required for *de novo *synthesis of telomeric repeats by telomerase [18,19]. Inhibition for distant region from template region has no effect on telomerase activity, so we chose the template region, GUC sequence, as a cleavage site [20,21].

Autexier [22]*et al *have proved that the functional area is located between 44 to 203 nt, in the experiment we cleave the template region located from 47 to 50 nt on hTR, and it should cause the significant reduction in telomerase activity.

In transacting gRZ.57, 16 nt was deleted from P4 stem, 6 base pairs in P1 were changed except G.U wobbling pair to meet the base pairing interaction between ribozyme and the substrate. The designed gRZ.57 exhibited cleavage activity.

We found that the extent of cleavage is about 70.4% in our research, no matter we increase the concentration of ribozyme or lengthen the time, it suggests that: (1) Ribozyme might conform differently and cannot combine with substrate. (2) Substrate was bound to Cs of the 3' of the ribozyme, not P1 stem. (3) A part of ribozyme-substrate complex adopts other conformation, and undergoes cleavage at a very low rate [23,24].

After eukaryotic expression plasmid of ribozyme was induced into 7402 cells and HCT116 cells, telomerase activity attenuated to 10% of that before, the telomerase activity of control cells doesn't change. This suggest that HDV ribozyme can cleave the hTR component as hammerhead ribozyme does, but its cleaving efficacy of is higher than that of hammerhead ribozyme [25].

Compared with L02 hepatocytes, bel 7402-RZ and HCT116-RZ cells mainly showed both Spontaneous apoptosis and blockage of cell cycle. In immortal cells, it has been shown that telomerase activity is associated with the cell cycle [26]. The highest telomerase activity is found in the S phase of cell cycle [27], whereas quiescent cells do not possess telomerase activity at a detectable level. Cancer cells escape senescence through both cell cycle checkpoint inactivation and the activation of telomerase. In addition to structural constraints[28], active telomerase is one possible factor to physically shield the telomeric G-rich singlestranded overhang. The presence of free G-rich single-stranded telomeric DNA within the nucleus was found sufficient to trigger cell cycle arrest in U87 glioblastoma cells and in human fibroblasts [29]. One might speculate that inhibition of telomerase might increase the probability that at some point in the cell cycle a free telomeric overhang becomes exposed to the nucleoplasm and could trigger cell cycle arrest or apoptosis.

It was also reported that the content of telomerase RNA in cells was not parallel to the telomerase activity [30]. In previous studies, hTR could be measured in cells, but there was no telomerase activity measured. Or, the hTR content in cells was measured high, but the telomerase activity was low. These results indicate that hTR is not the only determinant of telomerase activity. The catalytic protein subunits are believed to be the key determinant of telomerase activity [31].

In our northern, the uncut hTR decreased to 1/25 and 1/20 of the original in ribozyme transfected bel7402 cells and HCT116 cells respctively, while the telomerse activity drop to 1/10 and 1/8 respectively of the original. The results confirm the discrepancy of telomerase activity with telomerase RNA content.

Ribozyme-transfected bel7402 cells and HCT116 cells showed G1/G0 arrest and proliferation inhibition, and 75% cells showed apoptosis at 96 h. This is consistent with reduction of telomerase activity.

Our results suggest that diminution of telomerase can interfere with cancer cell growth and induce cell death, presumably through apoptosis. Emerging evidence revealed that telomerase activity is associated with increased cellular resistance to apoptosis [29,32,33]. Telomerase activity might therefore play some role in apoptosis-controlling mechanisms and inhibition of telomerase by ribozyme might impair this pathway.

## Conclusion

gRZ.57 we designed in the research is effective against the hTR, it is a promising agent for tumor therapy. HDV ribozyme may be used to cleave other molecules, such as viruses [34].

## Competing interests statement

The authors declare that they have no competing interests.

## Authors' contributions

YL has done part of the experiment, has drafted the manuscript and revised it. JG has supervised the experiment, have been involved in revising it critically for important intellectual content. DJ, YG did part of the experiment; MY has supervised the experiment. All authors read and approved the final manuscript.

## Authors' information

Yingying Lu, Ph.D., Associate professor, Department of Medicine, Beijing Friendship Hospital affiliated to Capital Medical University, Beijing, China 100050

Junchao Gu, Ph.D., Professor, Department of Medicine, Beijing Friendship Hospital affiliated to Capital Medical University, Beijing, China 100050
